# Brain BLAQ: *Post-hoc* thick-section histochemistry for localizing optogenetic constructs in neurons and their distal terminals

**DOI:** 10.3389/fnana.2015.00006

**Published:** 2015-02-04

**Authors:** David A. Kupferschmidt, Patrick A. Cody, David M. Lovinger, Margaret I. Davis

**Affiliations:** Section on Synaptic Pharmacology and In Vivo Neural Function, Laboratory for Integrative Neuroscience, National Institute on Alcohol Abuse and Alcoholism, National Institutes of HealthBethesda, MD, USA

**Keywords:** histochemistry, optogenetic neuroimaging, striosome and matrix compartments, tract-tracing, viral expression, Cre transgenic mouse

## Abstract

Optogenetic constructs have revolutionized modern neuroscience, but the ability to accurately and efficiently assess their expression in the brain and associate it with prior functional measures remains a challenge. High-resolution imaging of thick, fixed brain sections would make such *post-hoc* assessment and association possible; however, thick sections often display autofluorescence that limits their compatibility with fluorescence microscopy. We describe and evaluate a method we call “Brain BLAQ” (Block Lipids and Aldehyde Quench) to rapidly reduce autofluorescence in thick brain sections, enabling efficient axon-level imaging of neurons and their processes in conventional tissue preparations using standard epifluorescence microscopy. Following viral-mediated transduction of optogenetic constructs and fluorescent proteins in mouse cortical pyramidal and dopaminergic neurons, we used BLAQ to assess innervation patterns in the striatum, a region in which autofluorescence often obscures the imaging of fine neural processes. After BLAQ treatment of 250–350 μm-thick brain sections, axons and puncta of labeled afferents were visible throughout the striatum using a standard epifluorescence stereomicroscope. BLAQ histochemistry confirmed that motor cortex (M1) projections preferentially innervated the matrix component of lateral striatum, whereas medial prefrontal cortex projections terminated largely in dorsal striosomes and distinct nucleus accumbens subregions. Ventral tegmental area dopaminergic projections terminated in a similarly heterogeneous pattern within nucleus accumbens and ventral striatum. Using a minimal number of easily manipulated and visualized sections, and microscopes available in most neuroscience laboratories, BLAQ enables simple, high-resolution assessment of virally transduced optogenetic construct expression, and *post-hoc* association of this expression with molecular markers, physiology and behavior.

## Introduction

Viral-mediated expression of genetically encoded fluorescent proteins, light-activated channels and pumps, designer receptors, and signaling indicators allows for specific detection, manipulation, and interrogation of select neurons within heterogeneous populations both *in vivo* and *ex vivo*. However, viral expression of these constructs is highly variable, owing to factors such as viral titer, spread and transport, infection efficiency, toxicity, post-infection time interval, and promoter strength and specificity, in addition to the variability associated with anatomical differences and intracranial injection procedures. Given that small differences in somatic and terminal field expression of optogenetic constructs (e.g., in distinct but neighboring striatal subcompartments; Crittenden and Graybiel, 2011) can have important functional consequences, it seems essential to assess with high resolution the specificity and extent of their expression, and to associate this expression with molecular, physiological and behavioral measures in individual brain slices and animals. However, simple and efficient means to do so using conventional techniques are limited.

High-resolution imaging of thick brain slices of the sort commonly used in slice physiology experiments (>250 μm) would allow for efficient *post-hoc* assessment and association. However, the autofluorescence seen in thick sections often impedes imaging of the fluorescent signal of interest. This is particularly true in brain regions like the striatum due to its high lipid content and aldehyde-induced catecholamine autofluorescence (Carlsson et al., 1961; Falck et al., 1982; Clancy and Cauller, 1998). Several techniques and *post-hoc* transformations have been developed to address this problem. For example, autofluorescence can be separated based on spectrum, quenched using chemical treatments or reduced by imaging with multiphoton microscopy (Corrodi et al., 1964; Oliveira et al., 2010). Recent approaches such as Scale (Hama et al., 2011), ClearT (Kuwajima et al., 2013), CLARITY (Chung et al., 2013; Tomer et al., 2014) and SeeDB (Ke et al., 2013) achieve high-resolution imaging of deep tissue by clearing lipids in ways that retain the fluorescent signal. The ease, efficiency and wide-scale applicability of these techniques, however, are limited by the fact that they are relatively laborious, can result in soft and fragile tissue, can take several days to weeks to conduct, and can require costly and sophisticated imaging equipment (e.g., multi-photon confocal, serial array tomography, light sheet microscopy). Furthermore, any technique that requires high-resolution reconstruction to image whole brain or very thick tissue is limited by the requisite computing power (Osten and Margrie, 2013).

We describe and evaluate a method adapted from conventional histochemical techniques called Brain BLAQ (Block Lipids and Aldehyde Quench) to rapidly reduce autofluorescence in thick brain sections, enabling efficient axon-level imaging of neurons and their processes using standard epifluorescence microscopy. Unlike many methods, BLAQ can be performed *post-hoc* on thick sections commonly used in electrophysiological and optogenetic experiments with minimal time investment, inexpensive reagents, and simple optics. BLAQ can also be readily applied for meso-scale reconstruction after *in vivo* experiments using few sections that are easily manipulated, imaged, and archived. We demonstrate the utility of BLAQ by assessing the complex innervation patterns within striatum following viral-mediated transduction of various optogenetic constructs and fluorescent proteins in cortical pyramidal and midbrain dopaminergic neurons. The experiments we describe also revealed caveats and experimental factors that should be considered when using viral transduction and BLAQ histochemistry.

## Materials and methods

### Viruses

Adeno-associated viruses (AAVs) encoding Cre recombinase-dependent EGFP (AAV2/9.CAG.FLEX.EGFP.WPRE.bGH), GCaMP5 (AAV2/9.hSynap.FLEX.GCaMP5G(GCaMP3-T302L.R303P.D380Y).WPRE.SV40), GCaMP6 (AAV1.CAG.FLEX.GCaMP6s.WPRE.SV40) and tdTomato (AAV9.CAG.FLEX.tdTomato.WPRE.bGH) were purchased from University of Pennsylvania Vector Core. Viral Cre-dependent Channelrhodopsin 2 (ChR2; AAV5.EF1α.DIO.hChR2.H(134)R.mCherry) was obtained from University of North Carolina Vector Core.

### Mice

Emx1^Cre^ mice (B6.129S2-Emx1^tm1(cre)Krj^/J), which express Cre recombinase in the vast majority of cortical pyramidal cells (Gorski et al., 2002), were purchased from Jackson Labs (Bar Harbor, ME) and used for M1-targeted GCaMP/EGFP injections. Nr4a1-GFP mice (Tg(Nr4a1-EGFP)GY139Gsat/Mmucd), which express GFP in medium spiny neurons (MSNs) localized preferentially in striosomal compartments of the striatum (Davis and Puhl, 2011), were obtained from the Mutant Mouse Resource Center (Davis, CA) and crossed onto the C57Bl6J background for at least six generations. Offspring of Emx1^Cre^ and Nr4a1-GFP matings (B6;Emx1^Cre^;Nr4a1-GFP) were used for prelimbic (PrL Ctx) and infralimbic (IL Ctx) cortex-targeted tdTomato injections. Dopamine transporter-Cre (DAT^Cre^) mice (B6/CD1 mixed background; Tg (Slc6a3-cre)SG62Gsat/Mmucd), which express Cre recombinase in dopaminergic neurons (Gong et al., 2007), were used for ventral tegmental area (VTA)-targeted ChR2 injections. Male and female mice were used in each experiment. All animal work was performed in accordance with NIH policies for humane treatment of animals and approved by the NIAAA Animal Care and Use Committee.

### Virus injections

**Figure 1:**
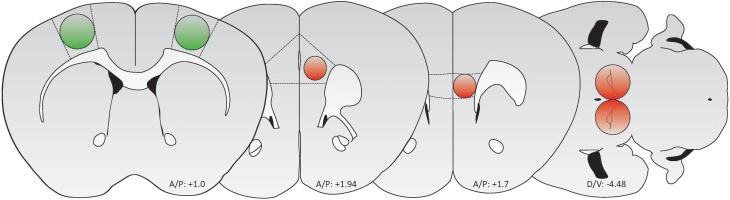
**Schematic of brain regions targeted for AAV-mediated transduction (Green: GFP/GCaMP; Red: tdTomato/mCherry)**. From left to right: M1 motor cortex, PrL cortex, IL cortex, and VTA. Adapted from Paxinos and Franklin (2012).

Under isoflurane anesthesia (1–5% in 0_2_), mice were stereotaxically injected with AAVs into various brain regions (Table 1; Figure 1). AAVs were manually injected using a 1 μL Hamilton syringe at 20–30 nL/min, and allowed to diffuse for 5 min following injection before syringe removal. Mice were at least 30 days of age at time of injection, and brains were analyzed 3–8 weeks post infection.

**Table 1:** **Injection sites, viral constructs, and mice used in the present experiments**.

**Site**	**Uni/Bilateral**	**Virus**	**Volume (nl)**	**Coordinates (Bregma/Dura)**	**Mouse**
M1	Bilateral	AAV2/9.CAG.FLEX.EGFP	300	A/P: +1.0	Emx1^Cre^
		AAV2.9.hSynap.FLEX.GCaMP5		M/L: ±1.7	
		AAV1.CAG.FLEX.GCaMP6s		D/V: −0.9	
PrL Ctx	Unilateral	AAV9.CAG.FLEX.tdTomato	80	A/P: +1.94	Emx1^Cre^; Nr4a1-GFP
				M/L: ±0.3	
				D/V: −1.5	
IL Ctx	Unilateral	AAV9.CAG.FLEX.tdTomato	80	A/P: +1.7	Emx1^Cre^; Nr4a1-GFP
				M/L: ±0.3	
				D/V: −2.15	
VTA	Bilateral	AAV5.EF1a.FLEX.ChR2.mCherry	300	A/P: −3.2	DAT^Cre^
				M/L: ±0.4	
				D/V: −4.48	

*Injection coordinates (relative to bregma/dura) and amounts are listed for each site. Full names of viral constructs and mouse strains are provided in the Materials and Methods Section*.

### Brain slice preparation

Mice were anesthetized with isoflurane, brains were removed, and 350-μm coronal brain slices (EGFP, GCaMP, tdTomato) and 250-μm horizontal slices (ChR2) were prepared using a vibratome. Coronal slices were equilibrated for 30 min at 33°C in 95% CO_2_/5% O_2_ bubbled with artificial CSF containing (in mM): 124 NaCl, 4.5 KCl, 2 CaCl_2_, 1 MgCl_2_, 26 NaHCO_3_, 1.2 NaH_2_PO_4_, and 10 glucose; slices were subsequently incubated in the same solution at 24°C. Horizontal slices were incubated at 24°C in a solution containing (in mM): 126 NaCl, 2.5 KCl, 2.4 CaCl_2_, 1.2 MgCl_2_, 25 NaHCO_3_, 1.2 NaH_2_PO_4_, 0.4 L-ascorbate, 20.1 HEPES, and 11 glucose. All slices were incubated for 1–6 h before fixation. Notably, the incubation solutions and durations reported here were used to accommodate electrophysiological, voltammetric and photometric recordings conducted on the slices imaged in this publication; however, Brain BLAQ can be performed immediately following slicing of perfusion-fixed or fresh brain tissue in 0.1 M phosphate buffer (PB) or phosphate-buffered saline (PBS).

### Reagents and antibodies

DAPI (4′, 6′-diamidino-2-phenlyindole, #D1306), Isolectin GS-IB_4_ (#I21411, #I21412) and 10X PBS (#70011) were purchased from Invitrogen (Carlsbad, CA). Sodium borohydride (#480886), 95% paraformaldehyde (#441244), ethanol (#E7148), Triton X-100 (#T8532) and Sudan Black B (#199664) were purchased from Sigma-Aldrich (St. Louis, MO). Chicken anti-GFP (1:2000; #AB13970) was purchased from Abcam (Cambridge, MA). Rabbit polyclonal anti-dsRed (1:1000; #632496) was purchased from Clontech (Mountain View, CA). Guinea pig anti-A_2A_ adenosine receptor (1:4000; A_2A_-GP-Af1000) was purchased from Frontier (Hokkaido, Japan). Rabbit anti-phospho-ERK (1:1000; #9101) was purchased from Cell Signaling (Beverly, MA). Rabbit anti-Iba (1:1000; #019-19741) was purchased from Wako (Osaka, Japan). AF488 goat anti-chicken (1:1000; #A11039), AF488 goat anti-guinea pig (1:1000; #A11073) and AF568 goat anti-rabbit (1:1000; #A11011) were purchased from Invitrogen.

### Brain BLAQ procedure

A list of required materials and a concise protocol for the Brain BLAQ procedure can be found in the Appendix (see Supplemental Data). Thick brain slices were fixed with 4% formaldehyde in 0.1 M PB or PBS. Fixative was initially at room temperature to preserve microtubules, which can be sensitive to cold fixation (Wallin and Stromberg, 1995); after 30 min, sections were transferred to 4°C for 12–24 h. All subsequent washes/incubations were performed in multi-well tissue culture dishes on an orbital shaker set to low speed. Sections were combined within wells to conserve antibody since the thick slices accommodated by this protocol could be easily ordered according to anatomical position following treatment.

Sections were washed for 1 h in PBST (PBS, 0.2% Triton X-100), and rinsed twice for 1 min in deionized water (diH_2_O) to remove ions and detergent. Slices were then incubated twice for 10 min in freshly prepared sodium borohydride (NaBH_4_; 5 mg/mL in diH_2_O) to quench aldehyde-induced catecholamine fluorescence and reduce free aldehyde groups. Following two 1-min rinses in diH_2_O, slices were incubated twice for 15 min at room temperature in filtered Sudan Black B solution (0.2% in 70% ethanol). Slices were washed twice for 30 min in room temperature PBS to mordant the stain and slow subsequent destaining. Additional quenching and blocking was occasionally performed if background fluorescence returned after extended washing.

Quenched and blocked brain sections were blue-black in color, rigid and easily manipulated with a paintbrush. The direct fluorescent signal was significantly attenuated (70–80%), and could be partially recovered by washing in PBST for 12–16 h. This attenuation was often advantageous since the signal from fluorescent reporters is frequently saturated near the injection site, distorting cellular-level resolution.

Immunostaining was used to amplify and stabilize the fluorescent signal in projections that are not easily detected because of the high background fluorescence or quenching by the preceding treatments. Slices were blocked using 5% BSA in PBST for 4 h, and incubated in primary antibody for 72 h at 4°C. Following four washes in PBST over a total of 16–24 h, slices were incubated in the appropriate Alexa-conjugated secondary antibody for 48 h at 4°C. Slices were then washed four times in PBST over a total of 16–24 h. DAPI (100 ng/mL) was added in some instances during the first of these PBST washes. Finally, slices were washed for 1 h in PBS to remove detergent and prevent destaining with storage.

Because the brain sections were robust and easily manipulated, they were often temporarily placed on a slide, imaged, and returned to the staining tray for subsequent processing or antibody stripping. To permit imaging from both sides, thick slices were either coverslipped in glass-bottom, multi-well plates, or mounted between two large coverslips separated by a spacer gasket made from a border of surgical tape (3M Transpore). Sudan Black leached into some mounting media with prolonged storage, leading to a darkening of the mounting medium and background fluorescence in the red channel. If this occurred, the background fluorescence could be easily reversed by removing the coverslip and rinsing the mounting medium away with PBS. Fluoromount Aqueous Mounting Medium (Sigma, F4680) did not cause significant leaching of the dye. Alternatively, slices could be stored in PBS at 4°C for at least 6 months with little apparent loss of signal.

**Figure 2:**
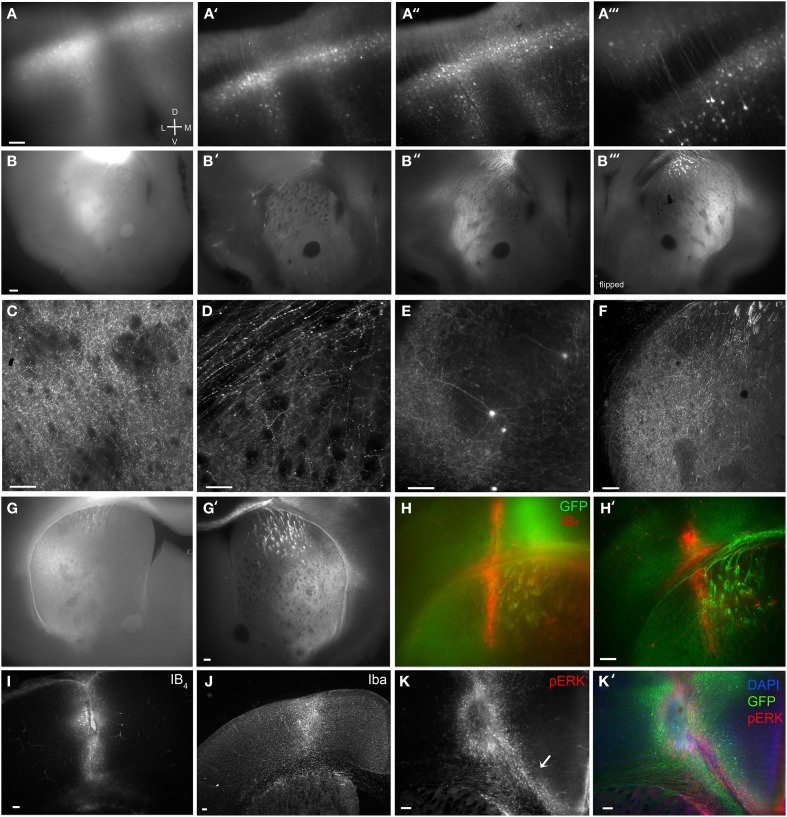
**Brain BLAQ detection of AAV-mediated GCaMP expression in M1 pyramidal cells and their projections in Emx1^Cre^ mice. (A–A″)** Expression of GCaMP5 at M1 injection site in a 350-μm-thick brain slice before treatment **(A)**, after quenching and blocking **(A′)**, and after immunostaining **(A″)**. **(A‴)** Lateral spread of infection from M1 to adjacent dorsal somatosensory cortex. **(B–B″)** GCaMP6s expression in dorsal striatum from the same section before treatment **(B)**, after blocking and quenching **(B′)**, and after immunostaining **(B″)**. **(B‴)** The opposite surface of the same section. **(C)** Axons from M1 projections terminating in the lateral striatal matrix after the full BLAQ protocol. **(D)** Axons in the external capsule and dorsal striatum. **(E)** Spread of M1 infection to distal regions of somatosensory cortex. **(F)** Striatal expression of GCaMP6s in a thin (50 μm), BLAQ-untreated, GFP-immunostained slice from an M1-injected Emx1^Cre^ mouse. **(G)** Striatal expression of GCaMP6s in a thick (350 μm), BLAQ-untreated, GFP-immunostained slice from an M1-injected Emx1^Cre^ mouse. **(G′)** GCaMP6s expression in an adjacent 350-μm slice from the same animal (processed in parallel with the slice in **G**) following the full BLAQ protocol. **(H,H′)** Thick adjacent slices from the same GCaMP6s-injected Emx1^Cre^ mouse immunostained for GFP and Isolectin GS-IB_4_ (IB_4_) without BLAQ treatment **(H)** and with BLAQ treatment **(H′)** to reveal the prior location of a fiber optic probe stereotaxically implanted into the dorsal striatum. **(I–K′)** M1 injection site identified by IB_4_ **(I)**, Iba **(J)** and pERK immunofluorescence (**K**, merged with GFP and DAPI in **K′**). Arrow in **K** indicates microglia streaming from the ventricular zone into the injection site. Scale bars, 100 μm.

Images were captured using Zeiss AxioVision LE 4.1 software with a Zeiss Axiocam on a Zeiss SteREO Lumar microscope equipped with standard DAPI, GFP and Cy3 filters, and a 1.5X telescoping zoom objective (12.5–150X, resolution 0.6 μm, z-precision 30 μm, field of view 23 mm, FWD 30 mm). This objective collected sufficient signal to image whole sections without the need to montage. Single axons were easily visible by direct GFP/RFP fluorescence at the maximum zoom with this objective. Background was subtracted in each channel and contrast enhanced for presentation. Digital gain was doubled to capture direct GFP fluorescence in striatum following blocking and quenching (Figure 2B′).

## Results

A typical AAV2/9-FLEX-GCaMP5 injection into M1 cortex of an Emx1^Cre^ mouse before Brain BLAQ exemplifies the poor fluorescence resolution in 350 μm-thick tissue commonly used for *ex vivo* recordings (Figure 2A). After quenching and blocking with sodium borohydride and Sudan Black, cells near the injection site were easier to resolve and many processes were visible without immunostaining (Figure 2A′); however, the remaining haze represents pyramidal cell collaterals that were no longer resolvable due to signal attenuation. After anti-GFP antibody amplification, individual deep and superficial pyramidal cells were clearly visible, as were many of their processes, even at low magnification (Figures 2A″,A‴).

As with the injection site, M1 projections to the striatum were poorly resolved before BLAQ (Figure 2B), weakly detected after blocking and quenching (Figure 2B′), but readily visualized after the full BLAQ protocol (Figure 2B″). The same section was flipped and imaged (Figure 2B‴), demonstrating the anatomical differences seen across a single 350 μm-thick section. At 150X magnification, individual processes were clearly visible within the matrix but very sparse in striosomes (Figure 2C). Striosomes avoided by M1 projections were most apparent in lateral and ventrolateral quadrants where sparse fibers contrasted with the black fiber bundles after BLAQ (Figure 2C). This pattern was observed in all 13 mice examined with little variability between subjects or the three viral constructs injected into M1. Our findings replicate earlier reports that motor cortex efferents preferentially target the matrix (Gerfen, 1984; Crittenden and Graybiel, 2011) and images from the Allen Institute mapping project showing sparse M1 projections to striosome-like regions within the striatum (http://connectivity.brain-map.org/). Individual axons within the corpus callosum and external capsule were also easily visualized after BLAQ (Figure 2D). Furthermore, sparse cells were clearly resolved in temporal somatosensory cortex (Figure 2E), suggesting infection of somatosensory axon terminals or viral transport.

Conventional fluorescence microscopy typically uses thin tissue sections to achieve sufficient antibody penetration and high-resolution imaging. Notably, the resolution and expression patterns of fluorescently labeled corticostriatal processes observed in thin (50 μm) GFP-immunostained, BLAQ-untreated sections from an Emx1^Cre^ mouse injected with GCaMP6s into M1 (Figure 2F) mimicked those seen following BLAQ treatment of thick (350 μm) sections (Figures 2A″,B″–E,G′,H′). Parallel processing of thick, GCaMP6s-expressing slices after GFP immunolabeling alone (Figure 2G; exposure time: 528 ms), relative to adjacent slices treated with the full BLAQ protocol (Figure 2G′; exposure time: 2.8 s), also showed the efficacy of BLAQ to reduce autofluorescence and signal saturation in thick tissue, and thereby allow higher resolution of corticostriatal afferents.

In addition to immunostaining for fluorescent proteins, we stained some sections to mark probe implant and virus injection sites. For example, to reveal the prior location of a fiber optic probe stereotaxically implanted into the dorsal striatum of a GCaMP6s-injected Emx1^Cre^ mouse, we co-stained thick (350 μm) sections for GFP and the microglial and perivascular marker, isolectin GS-IB_4_ (IB_4_; 1 μg/ml; Pennell et al., 1994). IB_4_ staining revealed the implant site and adjacent vasculature in neighboring slices processed in parallel either without BLAQ treatment (Figure 2H) or with BLAQ treatment (Figure 2H′). Both approaches effectively identified the implant site, but the full BLAQ protocol enabled higher resolution imaging of surrounding GCaMP6s expression. Similarly, staining for IB_4_ revealed the site of GCaMP6s injection in M1 (Figure 2I), while staining for the activated microglial marker, Iba, illuminated a larger region (Figure 2J). Immunostaining for phospho-ERK (pERK) was more sensitive at detecting activated microglia than isolectin GS-IB_4_ or Iba immunofluorescence, and marked numerous microglia streaming from the ventricles and into the injection site (Figure 2K, merged with GFP and DAPI in Figure 2K′; arrow in Figure 2K indicates streaming microglia). Immune responses are purported to be low with AAV constructs (Rogers et al., 2011) but may occur due to brain trauma, helper virus protein contamination, or endotoxin contamination (Nadeau and Rivest, 2000; Woodcock and Morganti-Kossmann, 2013), and are likely vastly under-reported in studies using intracranial surgery. BLAQ paired with microglia and pERK immunostaining can readily detect immune responses that may impact neuron function locally and downstream of the site of infection.

**Figure 3:**
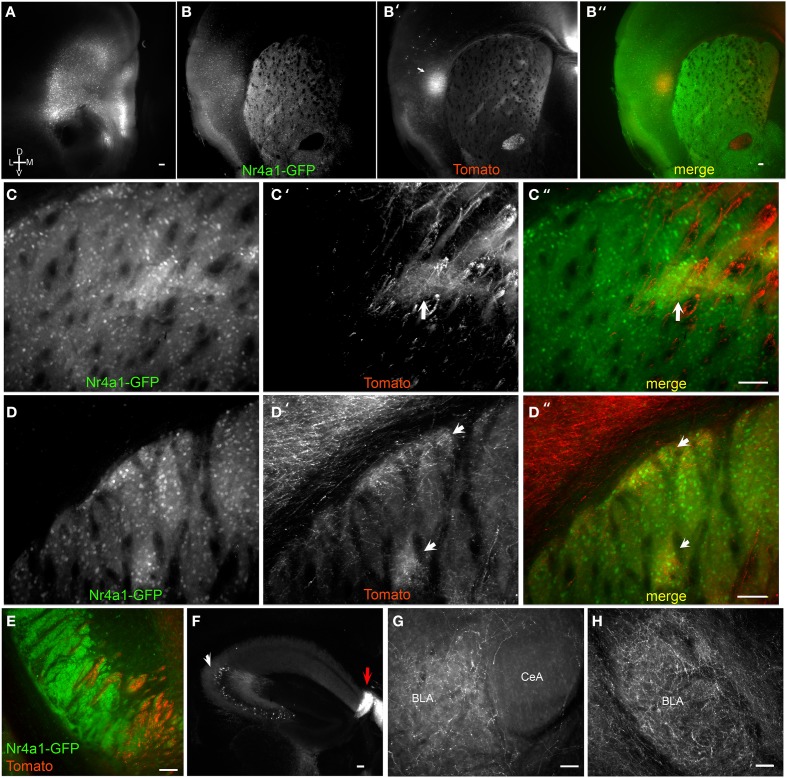
**Brain BLAQ detection of AAV-mediated tdTomato expression in mPFC pyramidal cells and their projections in Emx1^Cre^;Nr4a1-GFP mice. (A)** mPFC injection site in a 350-μm-thick brain slice after Brain BLAQ. **(B–B″)** Striosomes marked by Nr4a1-GFP expression **(B)**, mPFC tdTomato-expressing projections to striosomes **(B′)**, and overlay **(B″)**. **(C–C″)** Higher magnification of the dorsomedial striatum in **(B–B″)** stained for GFP **(C)** and tdTomato **(C′)**; overlay **(C″)**. **(D–D″)** Higher magnification of dorsolateral striatum in **(B–B″)** stained for GFP **(D)** and tdTomato **(D′)**; overlay **(D″)**. Arrows in **(C′,C″,D′,D″)** indicate patch-like structures. **(E)** tdTomato-expressing projections in the cerebral peduncles interdigitated with GFP from MSN afferents. **(F)** tdTomato expression in cells in CA3 of hippocampus (white arrow) and in dense fibers in the fasciola cinerea (red arrow). **(G–H)** mPFC projections targeting the basolateral amygdala (BLA) and avoiding the central nucleus of the amygdala (CeA). Scale bars, 100 μm.

While motor and sensory cortices preferentially target the striatal matrix, frontal cortical areas preferentially innervate striosomes (Gerfen, 1984). Medial prefrontal cortex (mPFC) to striosome projections were assessed with PrL/IL AAV9-FLEX-tdTomato injections into Emx1^Cre^; Nr4a1-GFP mice, which express GFP in MSNs localized preferentially in the striosomes (Davis and Puhl, 2011). Despite a small injection volume, viral spread was seen in some mice into adjacent orbital cortex (Figure 3A). Massive innervation of the claustrum and medial and lateral striosomes in the dorsal striatum was observed (Figures 3B–B″, arrow in Figure 3B′ indicates claustrum). Axons were clearly visible at 150X within these striosomes, adjacent to brightly fluorescent axon bundles in the dorsomedial striatum (Figures 3C–C″; arrows in Figures 3C′,C″ indicate cortical innervation of striosomes). Striosomes in the dorsolateral striatum and the subcallosal streak were also preferentially targeted by axons from the mPFC (Figures 3D–D″; arrows in Figures 3D′,D″ indicate cortical innervation of striosomes). Individual axons were discernible in both medial and lateral striosomes in thick sections after BLAQ. Different mice varied in their degree of striosome innervation, with those in which virus spread laterally from infralimbic to orbital cortex showing the most significant striosome selectivity.

Tomato-expressing mPFC projections were also seen in the medial cerebral peduncles, interdigitated with GFP-positive fibers arising from MSNs of Emx1^Cre^;Nr4a1-GFP mice (Figure 3E), but no somata were detected in the mesencephalon. While innervation of the hippocampal cornu ammonis (CA) regions and the dentate gyrus was faint, dense projections were observed in the fasciola cinerea, a structure contiguous with the dentate gyrus (Figure 3F, red arrow). Significant tomato expression was also seen in soma within CA3 (Figure 3F, white arrow) and occasionally in the amygdala, suggestive of viral transport. The amygdala also received dense mPFC projections (Figures 2G,H) that clearly avoided the central nucleus of the amygdala (Figure 3G).

**Figure 4:**
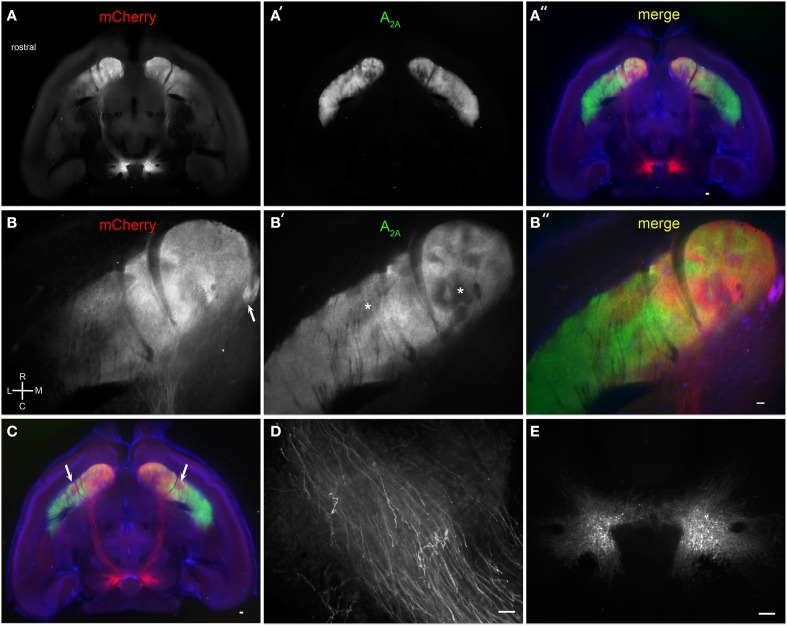
**Brain BLAQ detection of AAV-mediated ChR2-mCherry expression in VTA dopamine neurons and their projections in DAT^Cre^ mice. (A–A″)** Horizontal 350-μm-thick section at the level of the anterior commissure and ventral striatum stained for mCherry **(A)** and adenosine A_2A_ receptors **(A′)**; overlay with DAPI **(A″)**. **(B–B″)** Higher magnification of ventral striatum in **(A–A″)** stained for mCherry **(B)** and adenosine A_2A_ receptors **(B′)**; overlay with DAPI **(B″)**. Arrow in **(B)** indicates the Islands of Calleja. Asterisks in **(B′)** indicate heterogeneous structures in the NAc and presumed striosomes in the ventrolateral striatum. **(C)** More dorsal horizontal section through the ventral striatum of a different DAT^Cre^ mouse showing presumed bilateral striosomes targeted by VTA dopamine neurons (arrows). **(D)** Individual mCherry-expressing fibers in the medial forebrain bundle. **(E)** mCherry-expressing dopamine neurons at the injection site. Scale bars, 100 μm.

We next examined the efficacy of BLAQ in detecting dopaminergic fibers by tracing VTA projections to striatum labeled following AAV5-FLEX-ChR2-mCherry infusion into the VTA of DAT^Cre^ mice. Horizontal sections showed discrete subregions of VTA projections to the nucleus accumbens (NAc) and ventral striatum that diminished caudally and laterally (Figure 4A). Adenosine A_2A_ receptors are expressed by dopamine D2 receptor-expressing, indirect pathway MSNs (Svenningsson et al., 1999), but also show a heterogeneous matrix-like expression pattern in the ventral striatum (Figure 4A′). This matrix-like distribution was supported by A_2A_ staining in the Nr4a1-GFP mouse (M. I. Davis, unpublished observations). Some regions of segregation of A_2A_ receptor expression and VTA innervation were seen within the NAc and ventral striatum in all five mice examined (Figure 4A″, DAPI counterstain; higher magnification, Figures 4B–B″; more dorsal plane, Figure 4C). The Islands of Calleja, known to express the striosome marker FoxP2 (Takahashi et al., 2008), also received dense VTA projections (Figure 4B, arrow). Interestingly, putative striosome-like structures with dense VTA innervation (Figure 4C, arrows) localized to the same general region as those presented as a trio of islands in the ventrolateral striatum in the coronal plane that were nearly devoid of M1 innervation (Figure 2C). Higher (150X) magnification imaging of the medial forebrain bundle (Figure 4D) and single-cell detection at the injection site (Figure 4E) reiterate the utility of BLAQ for high-resolution fluorescence imaging of densely myelinated structures in thick tissue preparations.

## Discussion

We report a method adapted from conventional histochemical procedures called Brain BLAQ that enables efficient, high-resolution assessment of optogenetic construct expression in thick brain sections commonly used in electrophysiological and optogenetic experiments. Using standard epifluorescence microscopy of easily manipulated and visualized sections, and requiring minimal time investment and inexpensive reagents, Brain BLAQ provides investigators with a simple approach to reduce thick-tissue autofluorescence, enabling efficient *post-hoc* association of viral transduction with molecular markers and functional measures.

In addition to improved detection of virally transduced fluorescent constructs, BLAQ enables immunolabeling of many different native epitopes. We report here successful staining for adenosine A_2A_ receptors, the microglial marker Iba, and pERK, and have successfully stained (data not shown) for calbindin, cholecystokinin, parvalbulmin, tyrosine hydroxylase, and dopamine D1 receptors. Importantly, however, some antigens can be compromised by the BLAQ protocol, most likely by the brief 70% ethanol treatment. We found immunolabeling of cannabinoid-1 and mu-opioid receptors to be partially compromised by the BLAQ protocol. Notably, reducing the ethanol concentration or the duration of Sudan Black treatment can help retain antigenicity of certain epitopes (e.g., cannabinoid-1 receptors). However, the presence of ethanol in the Sudan Black B solution aids the penetration of Sudan Black B and antibodies in subsequent steps of the BLAQ protocol (Llewellyn-Smith and Minson, 1992); therefore, efforts to retain antigenicity may come at the expense of complete autofluorescence blocking and antibody penetration.

Brain BLAQ is not only suitable for use with standard epifluorescence microscopy, but is also amenable to laser scanning confocal microscopy and serial reconstruction. Confocal microscopy of BLAQ-treated tissue can resolve fluorescent signals over 150–200 μm deep with negligible loss of power (W. Shain and K. Trett, unpublished observations), so thick slices can be imaged from both sides to enable full reconstruction of the fluorescent signal. One important consideration noted during the development of BLAQ is tissue shrinkage. Brain sections shrunk by less than 5% when incubated for 5 min in the Sudan Black/ethanol solution, but closer to 30% when incubated for 30 min. As mentioned above for retaining antigenicity, using graded ethanol incubations or decreasing the duration of Sudan Black treatment will reduce shrinkage, but at the expense of antibody penetration and autofluorescence blocking. Although tissue shrinkage may be a concern for some applications where it is essential to retain precise three-dimensional morphology, it will be of little consequence for most applications (e.g., rapid assessment of overall viral transduction) if treated sections are assessed with reference to a rodent brain atlas (Simmons and Swanson, 2009).

We observed considerable variability in the extent and pattern of viral expression, particularly in downstream regions following AAV5-DIO-ChR2 targeting of VTA. For example, we noted sparse and highly variable VTA innervation of the basal amygdala across all DAT^Cre^ mice. Dense projections to the striosome-like (Millhouse, 1986; Davis and Puhl, 2011) intercalated cells of the amygdala were seen in only one of two DAT^Cre^ lines tested (second DAT^Cre^ line: Bello et al., 2011; two of two mice analyzed showed projections, data not shown). The variability between mice in VTA target regions may be individual mouse- and/or strain-specific, but underscores the need for validation of the extent and pattern of expression in each experimental animal (not only those injected for histology purposes), particularly when attempting to target small regions.

Although rare and somewhat controllable by selecting appropriate capsid/genome combinations (Cearley et al., 2008; Salegio et al., 2013), anterograde and retrograde AAV transport events can occur, can easily go undetected using serial thin sections unless each section is examined, and are nearly undetectable against background autofluorescence using conventional epifluorescence microscopy of thick sections. While our aim was not to systematically compare expression patterns and transport characteristics of each AAV serotype, BLAQ revealed somatic expression of the AAV9-FLEX-tdTomato construct in neurons innervating, or innervated by, the mPFC in mPFC-injected Emx1^Cre^ mice. Emx1^Cre^ mice express Cre in the vast majority of excitatory cells in the neocortex and hippocampus, and in sparse subpopulations in some ventral pallial structures (Gorski et al., 2002). We also observed significant local spread and probable transport of AAV2/9-FLEX-GCaMP5, but not AAV1-FLEX-GCaMP6, to other cortical regions and rarely to distal targets (e.g., basolateral amygdala) after M1 infection. Somatic expression was not detected in striatum of Emx1^Cre^ mice following M1 infection with any viral construct, suggesting against significant viral spread (despite modest Cre expression in striatum of Emx1^Cre^ mice), and distal transport of the VTA-injected viruses in DAT^Cre^ mice was not observed, although the latter may have been due to the absence of Cre-expressing cells in terminal regions. Significant local spread and transport can negatively impact experiments in which precise targeting of a single neuronal population is required, as with the expression of optogenetic constructs or designer receptors exclusively activated by designer drugs (DREADDs). Given that small differences in expression can result in meaningful functional differences, particularly in the striatum where subcompartments are believed to mediate very different aspects of movement, motivation, and pathology (Crittenden and Graybiel, 2011), unwanted viral transport and spread further exemplifies the need for a simple technique like BLAQ to evaluate the full extent of expression in each animal.

We demonstrated the utility of BLAQ using viral injections likely to differentially target striosome-matrix compartments within the striatum. Despite considerable evidence that striatal architecture is far more heterogeneous than the classical “direct” and “indirect” pathways, few groups examine the striosome-matrix subdivisions of the striatum and NAc core, or the column/wedge sub-architecture of the NAc shell (Jongen-Relo et al., 1993; Härtig et al., 2003; Gangarossa et al., 2013). By further clarifying the anatomical segregation of these functionally distinct striatal subregions, this work exemplifies the need to routinely associate higher resolution imaging of viral expression with physiological and behavioral measures obtained using optogenetics in individual mice. Brain BLAQ can serve as a useful tool to this end, enabling researchers to efficiently assess viral expression patterns at the axon-level in thick brain sections using standard microscopy available in most neuroscience laboratories.

### Conflict of interest statement

The authors declare that the research was conducted in the absence of any commercial or financial relationships that could be construed as a potential conflict of interest.
