# Supplementary material for: Brain BLAQ: Post-hoc thick-section histochemistry for localizing optogenetic constructs in neurons and their distal terminals
Source: Front Neuroanat. 2015 Feb 4;9:6. doi: 10.3389/fnana.2015.00006 (PMC4316788; doi:10.3389/fnana.2015.00006)
Supplement: Supplementary file 1 [file DataSheet1.DOCX]

**Appendix 1. Brain BLAQ Materials List.**

- Vibratome
- Orbital Shaker
- Formaldehyde (441244; Sigma-Aldrich)
- Multi-Well Cell Culture Plates (e.g. 24-well, Costar 3524, Corning Incorporated)
- 10X Phosphate-Buffered Saline (70011, Invitrogen)
- Triton X-100 (T8532, Sigma-Aldrich)
- Sodium Borohydride (480886, Sigma-Aldrich)
- Ethanol (95%, E7148, Sigma-Aldrich)
- Sudan Black B (199664, Sigma-Aldrich)
- Primary Antibodies (e.g. Chicken anti-GFP, AB13970, Abcam)
- Secondary Antibodies (e.g. AF488 Goat Anti-Chicken, A11039, Invitrogen)
- Slides (e.g. Superfrost Plus/Colorfrost Plus, Daigger)
- Coverslips (24x50 mm, ThermoScientific)
- Fluoromount (F4680, Sigma-Aldrich)
- Surgical tape (3M Transpore)
- Microscope (e.g. Zeiss SteREO Lumar microscope)

**Appendix 2. Brain BLAQ Procotol.**

- Place fresh thick slices in room temperature 4% formaldehyde made in 0.1 M PB or PBS; after 30 min, store slices at 4**°**C for 12-24 h.

*Note: Whole brains fixed by formaldehyde (e.g. by transcardial perfusion) and sliced at thicknesses ≤ 350 µm can similarly undergo the Brain BLAQ protocol.*

- In a multi-well tissue culture dish on an low-speed orbital shaker, wash slices for 1 h in PBST (PBS, 0.2% Triton X-100).
- Rinse slices twice for 1 min in diH_2_O.
- Incubate slices twice for 10 min in freshly prepared sodium borohydride (NaBH_4_; 5 mg/mL in diH_2_O).

*Note: NaBH_4_ causes bubbles to form in the well and on the brain slice surface.*

- Rinse slices twice for 1 min in diH_2_O.
- Incubate slices for 15 min in filtered Sudan Black B solution (0.2% in 70% ethanol).

*Note: Sudan Black B turns slices to a deep blue-black color. Native fluorescence signal is significantly attenuated (70-80%) by quenching and blocking, but can be partially recovered by washing in PBST for 12-16 h. Recovery of native signal can be facilitated by washing slices in PBST for longer durations at room temperature, and/or by using a higher concentration of Triton X-100 (e.g. 0.4%) in the PBST. Alternatively, additional quenching and blocking can be performed if unwanted background fluorescence returns after extended washing. Ethanol in Sudan Black B solution results in some tissue shrinkage and can compromise immunolabelling of certain antigens, so it is important to pre-assess the suitability of BLAQ for your particular experimental question.*

- Wash slices twice for 30 min in PBS.
- Incubate slices in 5% BSA in PBST for 4 h.
- Incubate slices in primary antibody for 72 h at 4°C.
- Wash slices four times in PBST for a total of 16-24 h at 4°C.
- Incubate slices in the appropriate fluorophore-conjugated secondary antibody for 48 h at 4°C.
- Wash slices four times in PBST for a total of 16-24 h at 4°C.

*Note: DAPI (100 ng/mL) can be added as a counterstain in the first of these PBST washes.*

- Wash slices for 1 h in PBS.

*Note: slices can be stored in PBS at 4°C for several months with little apparent loss of signal.*

- Mount slices using Fluoromount Aqueous Mounting Medium in either glass-bottom, multi-well plates with round coverslips, or between two large coverslips separated by a spacer gasket.

*Note: A bordering layer of 3M Transpore surgical tape serves as an effective spacer gasket.*

- Image slices using a conventional epifluorescence microscope, fluorescence stereomicroscope, or laser scanning confocal microscope.
